# Parameters of Cell Death and Proliferation of Prostate Cancer Cells with Altered Expression of Myosin 1C Isoforms

**DOI:** 10.1134/S1607672923700588

**Published:** 2024-01-07

**Authors:** E. S. Solomatina, E. N. Nishkomaeva, A. V. Kovaleva, A. V. Tvorogova, D. M. Potashnikova, A. A. Saidova

**Affiliations:** 1 Engelhardt Institute of Molecular Biology, Russian Academy of Sciences, Moscow, Russia; 2 Faculty of Biology, Moscow State University, Moscow, Russia; 3 Belozersky Research Institute of Physico-Chemical Biology, Moscow State University, Moscow, Russia

**Keywords:** myosin 1C, isoforms, prostate cancer, proliferation

## Abstract

Myosin 1C is a monomeric myosin motor with a truncated tail domain. Such motors are referred as slow “tension sensors.” Three isoforms of myosin 1C differ in short N-termed amino acid sequences, the functional differences between isoforms have not been elucidated. Myosin 1C isoform A was described as a diagnostic marker for prostate cancer, but its role in tumor transformation remains unknown. Based on data on the functions of myosin 1C, we hypothesized the potential role of myosin 1C isoforms in maintaining the tumor phenotype of prostate cancer cells. In our work, we showed that a decrease in the expression level of myosin 1C isoform C leads to an increase in the proliferative activity of prostate tumor cells.

*Abbreviations*: NLS, nuclear localization signal; PH, pleckstrin homology domain; TH1, tail homology domain; NM1, first nuclear myosin; B-WICH, chromatin remodeling complex B-WICH; FBS, fetal calf serum; TMRE, tetramethylrhodamine, ethyl ether.

## INTRODUCTION

Myosin 1C is the first monomeric myosin isolated from mammalian tissues and characterized by the presence of a three-domain structure typical of myosin motors with differences characteristic of this class of myosins that determine its special functions. The N-terminal motor domain of this protein (“head”) is capable of binding actin and ATP, and the rate of ATP hydrolysis is quite low, as a result of which this type of myosin functions as a tension sensor in the cell [1]. The region of the heavy chain with which the light chains are associated (“neck”) contains regulatory domains capable of binding calmodulin, calcium, and histone deacetylase, and also contains a nuclear localization signal. The C-terminal domain (“tail”) contains the pleckstrin homology (PH) domain and the tail homology domain (TH1) and ensures special functions of myosins [2].

In the cell, myosin 1C is represented by three isoforms differing only in their N-terminal regions: compared to the shortest isoform C, isoform B contains an additional 16 amino acid residues, and isoform A contains additional 35 amino acid residues, 10 of which are common to isoforms A and B [3]. Despite the high level of structural homology and the presence of an NLS sequence in the region common to all three isoforms, these proteins are characterized by a different nuclear-cytoplasmic ratio: isoform C is predominantly localized in the cytoplasm, whereas isoforms A and B are characterized by predominantly nuclear localization (isoform B is localized primarily in the nucleoplasm and nucleolus, while isoform A is not localized in the nucleolus, but is associated with nuclear speckles) [4, 5].

The majority of functional studies are aimed at studying the total fraction of myosin 1C, regardless of belinging to a certain specific isoform. Since this protein is able to interact with both lipids and actin, myosin 1C ensures direct interaction and generation of mechanical force between actin microfilaments and phosphoinositol of the cell membrane through the PH domain, participating in a number of intracellular processes. For example, myosin 1C is involved in maintaining the position of E-cadherin in cell contacts [6]. In addition, a decrease in the expression level of myosin 1C leads to disruption of the formation of tight junctions and cell migration of podocytes [7]. In neurons, myosin 1C is involved in the formation of lamellipodia, limiting their protrusion and regulating retrograde actin flow [8]. In adipocytes, myosin 1C accelerates the exocytosis of vesicles containing the glucose transporter GLUT4, stimulating glucose transport into adipocytes [9]. The functions of myosin 1C have been most fully studied in the model of sensory cells of the inner ear, where this protein is involved in the rapid closure of cation-selective channels [10].

Isoform A of myosin 1C is tissue-specific and is characteristic of normal tissue of the kidneys, adrenal glands, pancreas, and ovaries [11], whereas the other isoforms are expressed at similar levels in all tissues and organs. It was proposed to use this isoform as a tumor-specific marker, since its overexpression is characteristic of prostate cancer cells and prostate tumor tissue of the TRAMP model mice [11]. The analysis of the expression of isoform A in prostate cancer cell lines showed that, using real-time PCR, it is possible to detect an increase in the level of mRNA expression of this isoform even in samples with a small number of cells or in samples with a large amount of the stromal component [12]. In addition, the level of expression of isoform A is significantly increased in clinical samples of prostate cancer and makes it possible to distinguish not only the tumor process from reactive benign prostatic hyperplasia but also the stages of the tumor from each other, which opens up new prospects for its use as a diagnostic and prognostic marker [13]. In this work, we hypothesized that exogenous changes in the expression level of myosin 1C may affect the balance of death parameters in prostate cancer cell lines. To test this hypothesis, we derived model prostate cancer cell lines with inducible expression of myosin 1C isoforms A and C (increased expression model) and used siRNA to myosin 1C isoforms A and C (reduced expression model). Using model cell lines PC-3 and LNCaP with increased and decreased expression of myosin 1C isoforms, respectively, we analyzed the proliferative activity of cells using the Ki-67 marker and spontaneous cell death using the annexin V marker and the voltage-dependent dye TMRE.

## MATERIALS AND METHODS

### Cell Lines

Human prostate cancer cell lines PC-3 (CRL-1435) and LNCaP (CRL-1740) were obtained from the American Type Culture Collection (ATCC, United States). Cells were cultured in DMEM:F12 medium (1 : 1) supplemented with 10% fetal calf serum (HyClone, United States), 0.01 g/L gentamicin, 0.3% amphotericin B, and 584 g/L L-glutamine (PanEco, Russia). Passaging was performed every 3 days in a ratio of 1 : 5 using a 0.05% trypsin-EDTA solution (PanEko, Russia).

### siRNA Transfection

Using the online tool https://rnaidesigner.thermofisher.com/rnaiexpress/, siRNAs for knockdown of isoform A and for knockdown of all three isoforms of myosin 1C were written. The miRNA sequences are shown in Table 1. The synthesis and annealing of sequences was carried out at DNK-Sintez (Russia). Transfection of siRNA was performed using the Turbofect transfection agent (Thermo Fisher Scientific, United States) according to the manufacturer’s instructions at a siRNA concentration of 200 pmol/μL in knock-out DMEM medium (Thermo Fisher Scientific, United States). Physiological effects in cells were studied after 92 h of exposure. Cells were removed using a 0.05% trypsin-EDTA solution and culture medium and centrifuged at 750*g* for 5 min. During any experiment, part of the cells were taken for further verification of expression suppression.

**Table 1.** Sequences of siRNA for knockdown of myosin 1C isoforms

Myosin 1C isoform	Sequence
siMYO1CA	AUGAACCACGCGGAUGAUCUCCdTdT
siMYO1CC	CCUAUCGCCGCAAAUAACGAGCdTdT

### RNA Extraction and Real-Time PCR

To determine the expression level of myosin 1C isoforms, total RNA was isolated from dried sediments of PC-3 cells using a commercial RNeasy Mini Kit (Qiagen, United States) according to the manufacturer’s recommendations. The RNA concentration was measured and its quality was assessed using a NanoPhotometer spectrophotometer (Implen, Germany).

RNA (1 μg) was taken into the reverse transcription reaction, which was carried out using a commercial MMLV RT kit for cDNA synthesis (Evrogen, Russia) according to the manufacturer’s recommendations. When performing real-time PCR, the nonspecific DNA intercalating dye SYBR Green I (iTaq reagent kit, Bio-Rad, United States) was used. The working concentration of each primer was 10 pmol. Three technical replicates were used for each pair of primers. Primers for real-time PCR were synthesized at OOO Evrogen (Russia), the sequences are given in Table 2, the primers for the reference genes are described in [12]. The primary analysis of real-time PCR results was performed as described in [14].

**Table 2.** Primers for real-time qPCR

Myosin 1C isoform	Primer nucleotide sequence 5'–3'	Temperature of annealing, °C
A	for: GCTCGAGGCGCTGCAAGTGGAGCTGG	65
	rev: CGCTAGCTCACCGAGAATTCAGCCGTGG	
C	for: GTACAGCGTGCGGACAATAAGC	62
	rev: CCTTGGTGATGAGCAGCTCC	

### Obtaining Stable Cell Lines with Inducible Expression of Myosin 1C Isoforms

Using the RNA template isolated from PC-3 cells using the Trizol method, cDNA was obtained using reverse transcriptase (Maxima Reverse Transcriptase, Thermo Fisher Scientific, United States). Sequences were generated on the cDNA template using Q5 High-Fidelity DNA Polymerase (NEB, United States). For further ligation, *Xho*I and *Nhe*I restriction endonuclease site sequences were added to the primers. Ligation of the obtained sequences into the pBlu2SKM auxiliary vector (hereinafter, pSK) was performed at the *EcoR*V site. The ligation reaction in pSK was performed at a ratio of 1 : 3 (50 ng of vector + 150 ng of inserts) using a Rapid DNA Ligation Kit (Thermo Fisher Scientific, United States). Transformation was performed in *E. coli–*XL1–Blue by electroporation. Ligation was assessed by test restrictions of plasmids at the *Hind*III restriction site. Clones selected on the basis of restriction tests were purified using HiPure Gel DNA Mini Kit columns (Magen, China). The integrity of the *Xho*I and *Nhe*I restriction sites, as well as the absence of point mutations, was confirmed by Sanger sequencing performed by OOO Evrogen (Russia). A 5-kb PCR product was generated from the obtained vectors using Invitrogen Platinum SuperFi II DNA Polymerase (Thermo Fisher Scientific, United States) according to the manufacturer’s protocol. Using NEBuilder HiFi DNA Assembly (NEB, United States), the 15.5-kb pSLIK Flag vector with inserts of myosin 1C isoforms A and C was assembled from two additional fragments. Transformation was carried out in *Stbl E. coli* by electroporation. Clones were assessed using test restrictions of plasmids at the *BamH*1 and *Nde*I restriction sites. Lentiviral particles were assembled using pSLIK(Flag)-MYO1C-iso A/C plasmids and VSV-G and pCMV-dR8.2 auxiliary plasmids in HEK293T cells. After transfection, the conditioned medium was collected every 24 h for 3 days; after centrifugation, the supernatants were filtered through a 0.22-μm filter. Stable lines were selected using the selective antibiotic G418 (Thermo Fisher Scientific, United States) with a working concentration of 0.0005 g/mL. The increase in the expression level of these genes was verified using Western blot analysis. Physiological effects in cells were studied after 72 h of expression induction in the presence of 0.001 mg/mL doxycycline.

### Western Blot Analysis

The cells were removed with a 0.05% trypsin-EDTA solution and centrifuged at 750*g* for 5 min, the supernatant was removed, and the Eppendorf tubes with pelleted cells were placed on ice. Cells were lysed by adding 100 μL of RIPA buffer containing PIC, PHIC II, and PHIC III (Merck, United States). Samples were incubated on ice for 30 min and then centrifuged for 20 min at 4°C at 12  000*g*. Proteins were separated by electrophoresis in 7.5% polyacrylamide gel; before application, samples were incubated for 15 min at 98°C. Transfer to a nitrocellulose membrane (Thermo Fisher Scientific, United States) was carried out at 80 mA for 70 min. After transfer, the membrane was incubated for 30 min in a 5% milk solution (Nonfat Dry Milk, Cell Signaling, United States). Next, the membrane was incubated for 8 h at 4°C in a solution of primary antibodies to the FLAG peptide (clone M2, Sigma, United States) or to the total myosin 1C (clone EPR14771, Abcam, United States) in a 5% milk solution and then incubated with secondary anti-mouse IgG antibodies (clone 7076, Cell Signaling, United States). Pierce™ ECL Western Blotting (Thermo Fisher Scientific, United States) was used for development.

### Cell Cycle Analysis

Cell cycle analysis was performed by flow cytometry using standard staining with propidium iodide (Amresco, United States) as described previously [15]. Data were recorded using a FACSAria SORP instrument with FACSDiva 6.2 software (BD Biosciences, United States).

### Proliferative Potential Analysis

The proliferative potential was analyzed by flow cytometry using staining for the proliferating cell marker Ki-67. The collected cell suspension was fixed by incubating with 1% paraformaldehyde (Merck, United States) for 10 min at room temperature, washed once with PBS, and permeabilized with a ×1 Perm II solution (BD Biosciences, United States) according to the manufacturer’s recommendations. After washing with PBS, the cells were stained with anti-Ki-67-FITC antibodies (clone B56, BD Biosciences, United States) in accordance with the manufacturer’s recommendations. The polygon of Ki-67+ cells in the preparation was distinguished by comparison with the negative control (unstained suspension of the same cells).

### Cell Death Assay

Spontaneous cell death was assessed by flow cytometry using the voltage-dependent TMRE dye (Thermo Fisher Scientific, United States) and annexin V-FITC (BioLegend, United States) to detect externalized phosphatidylserine. The protocol for co-staining with TMRE and annexin V was described previously [16]. Data were recorded and analyzed using the FACSAria SORP instrument with FACSDiva 6.2 software.

### Data Analysis and Presentation

Flow cytometry data were analyzed using Diva 6.2.1 software (BD Biosciences, United States). In analyzing the data, subpopulations corresponding to different cell cycle phases were distinguished automatically using the ModFit LT 3.3 software. Plots were constructed and analysis was performed using the GraphPad Prism 8 software. Differences were statistically analyzed using the Kruskal–Wallis test. Differences were considered statistically significant at *p* < 0.05.

## RESULTS AND DISCUSSION

### Expression Level of Myosin 1C Isoforms in Model Cell Lines

**Fig. 1.**
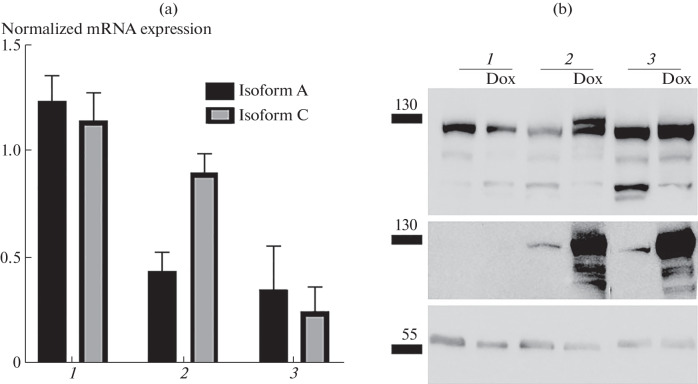
Expression of myosin 1C isoforms in model cell lines. (a) Level of mRNA expression of isoforms A and C of myosin 1C in the control (1), with the addition of siRNA to isoform A of myosin 1C (2), and with the addition of siRNA to isoform C of myosin 1C (3). Mean values and standard deviations are shown; data are normalized to the reference genes *YWHAZ*, *GAPDH*, and *HPRT1*; five independent experiments are shown. (b) Expression of isoforms A and C of myosin 1C in LNCaP cells in control and with the addition of doxycycline (Dox). I—staining with antibodies to pan-myosin 1C, II—staining with antibodies to the FLAG tag, III—staining with antibodies to α-tubulin; 1—control, 2—cell line FLAG-Myo1C-isoA, 3—cell line FLAG-Myo1C-isoC.

The effectiveness of siRNAs to myosin 1C isoforms was assessed by real-time PCR. The use of siRNA to isoform A made it possible to reduce the expression level of this isoform in PC-3 cells by 2.9 times, whereas the expression level of isoform C relative to the control did not change significantly (Fig. 1a), which indicates the specificity of siRNA to isoform A. The use of siRNA to isoform C made it possible to reduce the expression level of isoform A by 4.2 times, and the expression level of isoform C decreased 4.6 times, since siRNA to isoform C suppresses the expression of both the shorter isoform C and the longer isoform A.

The effectiveness of the doxycycline-induced increase in the expression level of myosin 1C isoforms in LNCaP model cell lines was confirmed by Western blot analysis (Fig. 1b). In this work, we generated LNCaP cell lines with inducible expression of isoform A or isoform C, which were fused to a FLAG tag. Using antibodies to the FLAG tag, we detected a specific increase in the expression level of both isoform A and isoform C of myosin 1C in response to addition of doxycycline. Further, for convenience, the lines were  designated as follows: line PC-3 with knockdown of isoform A—si_MYO1C_isoA, line with knockdown of isoform C of myosin 1C—si_MYO1C_isoC, line LNCaP with inducible overexpression of isoform A—FLAG-Myo1C-isoA, and line with inducible overexpression of isoform C—FLAG-Myo1C-isoC.

### Basic Parameters of the PC-3 Cell Line in Normal State and at Reduced Expression of Myosin 1C

**Fig. 2.**
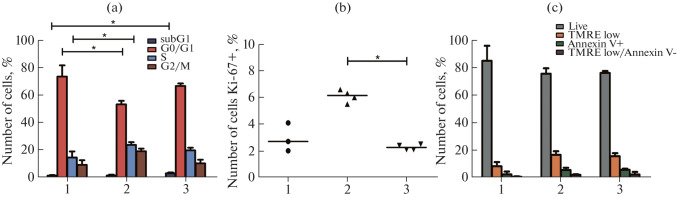
Main parameters of proliferation and cell death in the PC-3 prostate cancer cell line in control (1), with downregulation of myosin 1C isoform A (2), and with downregulation of myosin 1C isoform C (3). (a) Histograms of cell distribution over the cell cycle. (b) Abundance of Ki-67+ cells in the samples. (c) Histograms of cell distribution by stages of cell death. Three independent experiments are presented, * significant differences between groups, *p* < 0.05, Kruskal–Wallis test, Dunn’s post-test.

The main parameters of proliferation and cell death in the model prostate cancer cell line PC-3 and in its modifications were assessed by flow cytometry. The distribution of cells by the cell-cycle phases was assessed using the standard propidium iodide staining. The assessment of the distribution by cell-cycle phases revealed significant differences between the original PC-3 culture and the culture with isoform C knockdown: the number of cells in the G0/G1 phase was significantly reduced, and the number of cells in the S phase was significantly increased in the si_MYO1C_isoC line. When isoform A was knocked down in the si_MYO1C_isoA line, we detected a significant increase in the sub G1 population, which included hypodiploid cells and apoptotic cells with degraded DNA (Fig. 2a).

To analyze the proliferative potential of the PC-3 cell line and its modifications, we performed staining for the marker of proliferating cells Ki-67 (Fig. 2b). The percentage of Ki-67+ cells was 2.93 ± 1.07% for the control PC-3 culture, 6.10 ± 0.46% for the si_MYO1C_isoС culture, and 3.28 ± 0.21% for the PC-3 si_MYO1C_isoA culture. The percentage of proliferating cells was significantly increased in the cells with knockdown of isoform C compared to knockdown of isoform A. The analysis of spontaneous cell death (Fig. 2c) showed that the percentage of living cells with a high TMRE fluorescence intensity and without annexin V staining was 85.20 ± 11.01% for the control culture PC-3, 75.80 ± 4.00% for the si_MYO1C_isoС line, and 76.40 ± 1.41% for the si_MYO1C_isoA line. The differences in the representation of all distinguished populations of living and dying cells were not significant. Thus, knockdown of myosin 1C and separately its isoform A does not affect the process of spontaneous cell death in the PC-3 culture. However, knockdown of the isoform C of myosin 1C activates the proliferative activity of PC-3 cells: more cells compared to the control enter the S phase and more cells carry the Ki-67 marker compared to knockdown of separate isoform A.

### Basic Parameters of the LNCaP Cell Line in Normal State and at Induced Expression of Myosin 1C

**Fig. 3.**
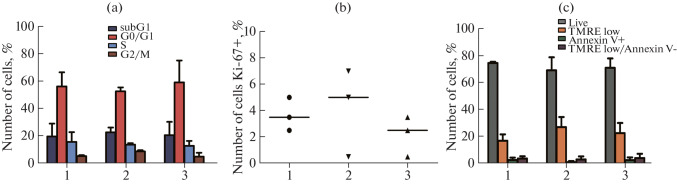
Main parameters of proliferation and cell death in the prostate cancer cell line LNCaP 3 in control (1), with overexpression of myosin 1C isoform A (2), and with overexpression of myosin 1C isoform C (3). (a) Histograms of cell distribution over the cell cycle. (b) Abundance of Ki-67+ cells in the samples. (c) Histograms of cell distribution by stages of cell death. Three independent experiments are presented, * significant differences between groups, *p* < 0.05, Kruskal–Wallis test, Dunn’s post-test.

The main parameters of proliferation and cell death in the prostate cancer cell line LNCaP and in its modifications were assessed by flow cytometry. Staining for the distribution by cell-cycle phases with propidium iodide did not show significant differences between the original LNCaP culture and its modifications (Fig. 3a).

The number of Ki-67+ cells was 0.73 ± 0.25% for the control LNCaP culture, 0.83 ± 0.67% for the LNCaP FLAG-Myo1C-isoC culture, and 0.43 ± 0.31% for the LNCaP FLAG-Myo1C-isoA culture. No significant differences in the number of proliferating cells between the experiment and the control were found (Fig. 3b). The analysis of spontaneous cell death in the control LNCaP culture and its modifications (Fig. 3c) showed that the percentage of living cells was 74.47 ± 0.90% for the control, 69.07 ± 9.66% for the LNCaP FLAG-Myo1C-isoC culture, and 70.90 ± 6.99% for FLAG-Myo1C-isoA line. The differences in the representation of all distinguished populations of living and dying cells were not significant. Thus, the induced expression of myosin 1C isoforms does not affect the processes of cell proliferation and spontaneous cell death in the LNCaP culture.

We have shown that changes in the expression level of myosin 1C (either increase or decrease) do not affect the percentage of spontaneously dying cells in prostate cancer lines. At the moment, the relationship between myosin 1C and various types of cell death is analyzed in a small number of studies. For example, the absence of functional myosin 1C leads to disruption of autophagy; this process is apparently associated with the involvement of myosin 1C in the proper functioning of the vesicular compartment of the cell [17].

Uncontrolled cell proliferation is one of the main signs of oncological transformation [18]. For different subtypes of prostate cancer and tumors in general, the induction of proliferative activity can occur in different ways. It is known that an increase in proliferative activity can be achieved by increasing the expression of growth factor receptors or the presentation of receptors on the cell surface, due to mutations that lead to constitutive activation of growth factor signaling pathways, due to disruption of the mechanisms that control proliferation, as well as due to changes affecting disturbances in contact inhibition [19]. Since myosin 1C is involved in the regulation of vesicular transport and exocytosis of transmembrane proteins, it can influence the presentation of growth factor receptors on the cell surface [20]. In addition, myosin 1C is involved in the creation and maintenance of intercellular adhesion. Therefore, the suppression of its expression can lead to impaired contact inhibition of epithelial cells [6].

We have shown that a decrease in the expression level of myosin 1C isoform C leads to an increase in the proliferative activity of PC-3 culture cells, with significantly more cells being in the S phase of the cell cycle. At the same time, knockdown of only isoform A does not affect proliferative activity, but leads to a significantly greater accumulation of cells in the sub-G1 phase, which indicates the importance of this isoform for the survival of tumor cells.
